# Multiplexed Digital Characterization of Misfolded Protein Oligomers via Solid-State Nanopores

**DOI:** 10.1021/jacs.3c09335

**Published:** 2023-11-16

**Authors:** Sarah E. Sandler, Robert I. Horne, Sara Rocchetti, Robert Novak, Nai-Shu Hsu, Marta Castellana Cruz, Z. Faidon Brotzakis, Rebecca C. Gregory, Sean Chia, Gonçalo J. L. Bernardes, Ulrich F. Keyser, Michele Vendruscolo

**Affiliations:** † Cavendish Laboratory, Maxwell Centre, Department of Physics, University of Cambridge, Cambridge CB3 0HE, U.K.; ‡ Centre for Misfolding Diseases, Yusuf Hamied Department of Chemistry, University of Cambridge, Cambridge CB2 1EW, U.K.; § Bioprocessing Technology Institute, Agency for Science, Technology and Research (A*STAR), Singapore 138668

## Abstract

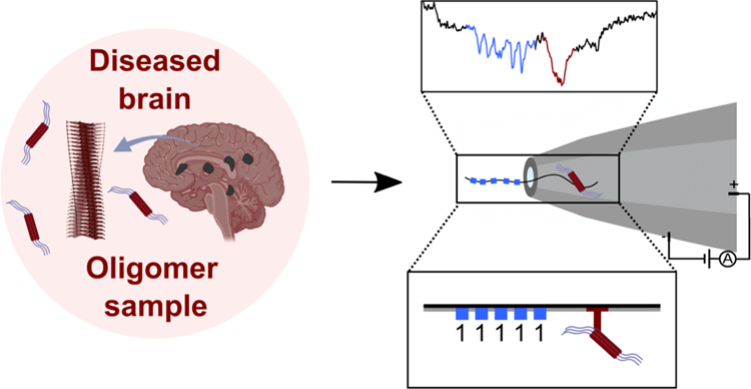

Misfolded protein oligomers are of central importance in both the diagnosis and treatment of Alzheimer’s and Parkinson’s diseases. However, accurate high-throughput methods to detect and quantify oligomer populations are still needed. We present here a single-molecule approach for the detection and quantification of oligomeric species. The approach is based on the use of solid-state nanopores and multiplexed DNA barcoding to identify and characterize oligomers from multiple samples. We study α-synuclein oligomers in the presence of several small-molecule inhibitors of α-synuclein aggregation as an illustration of the potential applicability of this method to the development of diagnostic and therapeutic methods for Parkinson’s disease.

## Introduction

The presence of misfolded protein oligomers is associated with the onset and progression of several neurodegenerative disorders, including Alzheimer’s and Parkinson’s diseases.^1,2^ These species are likely to form because many proteins may be present in the cell at supersaturated concentrations, making them prone to aggregation, and driving the interconversion between functional states and aberrant self-assembled multimerized states.^3,4^ To prevent this outcome, under normal conditions, the protein homeostasis systems including molecular chaperones and the ubiquitin-proteasome and endosomal-lysosomal degradation pathways^5,6^ ensure the correct folding and complexing of proteins and removal of aggregates.^7,8^ The metastable proteome becomes increasingly unstable however, as the body ages and experiences stresses, concomitant with these maintenance pathways becoming less efficacious.^9,10^ This leads to uncontrolled protein aggregation and the accumulation of these misfolded oligomers, eventually converting to highly ordered polymeric fibrils.^1,2,11^

**Figure 1:**
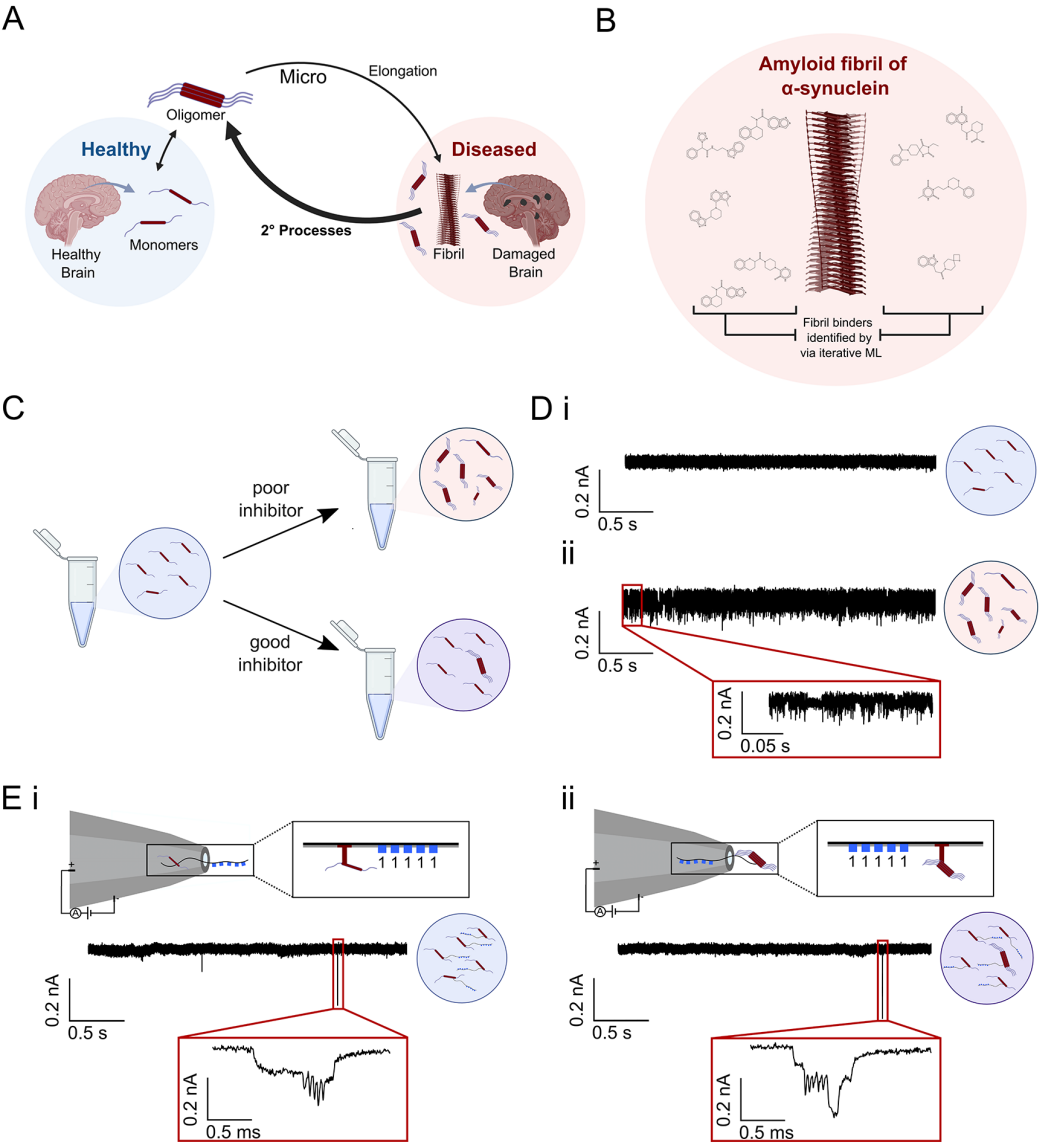
Schematic illustration of the process of αS oligomer formation in Parkinson’s disease, of its inhibition by compounds that can block secondary nucleation,^39^ and of the method reported here to measure the efficacy of these compounds, which is based on DNA nanostructures. (A) Age-related progressive impairment of the protein homeostasis system leads to the aberrant misfolding and aggregation of αS into toxic oligomeric species, which eventually convert to amyloid fibrils. These fibrils are observed as the primary constituents of Lewy bodies, a hallmark structure observed in brain cells of patients suffering from the disease. Fibrils can act as a catalyst for further oligomer formation via secondary processes, such as secondary nucleation from catalytic sites on the fibril surface and fragmentation of the fibrils into smaller species. Secondary processes are the key generators of oligomeric species. (B) Structure-based iterative machine learning strategy composed of docking simulations followed by cycles of active machine learning was employed in a parallel work by the authors to identify secondary nucleation inhibitors.^39^ I3.08 from that work is used here as a tool compound here. (C) Oligomer inhibitors have different efficacies, which have previously been challenging to establish, given how difficult oligomers are to measure. (D) Previous approaches to oligomer measurement in nanopores have attempted to measure protein levels in the absence of any tagging methods, which is a difficult task prone to error given how challenging individual oligomer translocations are to reliably differentiate from each other and from monomer. Monomer (i) and heavily oligomerized (ii) samples are shown as examples in an uncoated pore with a diameter of ∼15 nm. Oligomers cannot be readily probed at a single-molecule level via this approach, meaning that only bulk levels can be measured. (E) A novel oligomer measurement approach employing unique DNA nanostructure barcoding of each particle in a sample enables both single-molecule resolution of oligomers and multiplexing of samples, delivering improved metrics of inhibitor efficacy and increased throughput. (i) Monomeric protein with an attached barcode exhibits no adjacent spike, as the nanopore diameter has been tailored so that monomers do not generate a signal. (ii) Lightly oligomerized sample exhibits a clear spike in association with the unique barcode. The barcoded protein can enter the pore in either orientation (barcode first or protein first).

Numerous neurodegenerative diseases are thought to result in part because of this, as aggregates accumulate and interfere with crucial neuronal functions.^1,2,12,13^ The aggregation of α-synuclein (αS), for example, is associated with the initial neurodegenerative processes underlying Parkinson’s disease, in which αS aggregates, and misfolded oligomers in particular, exhibit various mechanisms of cellular toxicity.^14−19^ Therapeutic efforts directed at this area have not yet resulted in approved drugs.^20^ In part this is because they are based on readouts related to αS fibrils, which are the end point of the aggregation process. These highly ordered structures are thought to be largely inert in terms of neuronal toxicity, although they can catalyze the formation of further oligomers via a process termed secondary nucleation^21−26^ (Figure 1A). To date, most investigations into the aggregation process rely on the detection of fibrils using amyloid-binding dyes, such as thioflavin T (ThT), that fluoresce strongly upon binding to fibrils. This approach, however, does not provide a direct measure of the oligomers present, the population of which varies according to the mechanism of aggregation.^27,28^

A promising therapeutic strategy is the blocking of secondary nucleation (Figure 1B), which is a key accelerator of oligomer production.^29,30^ Specifically targeting oligomer-producing steps is essential. If fibril elongation were to be inhibited for example, this would slow the formation of end point fibril but increase the population of misfolded oligomers by shifting the aggregation pathway more strongly toward secondary nucleation (Figure 1A,C).^27^ Previous work has shown methods of isolating specific mechanisms of aggregation and their respective rates experimentally, and subsequently inferring the oligomer populations at a given time via fitting to an analytical model of the aggregation process.^21,28^ Theoretical predictions were previously experimentally validated by taking samples during the aggregation process, tracked via ThT, and separating by size exclusion chromatography (SEC) before measuring the monomer equivalent oligomer concentration in each lyophilized sample via mass spectrometry (MS) or enzyme-linked immunosorbent assays (ELISA).^28,31^ While this is a valid strategy, it is hampered by low throughput and technical challenges in the implementation. Therefore, there remains a need to experimentally probe the oligomer population in a nondisruptive and higher-throughput manner to determine the size distributions of the oligomer population over time at single particle resolution.^32^

Thus far, single-molecule techniques have shown promising results in characterizing oligomer distributions.^32^ For example, confocal two-color coincidence detection (TCCD),^33^ fluorescence correlation spectroscopy (FCS) measurements,^34^ single-molecule total internal reflection fluorescence (TIRF) imaging,^35^ single-molecule spectrally resolved points accumulation for imaging in nanoscale topography (sPAINT),^36^ atomic force microscopy (AFM),^37^ and micro free-flow electrophoresis (μFFE)^38^ have all allowed study of oligomer distributions under near-physiological conditions. Additionally, it has been shown that using μFFE one can ascertain oligomer populations in the presence of specific secondary nucleation inhibitors.^38,39^ One major limitation of these approaches, however, is the low throughput at which molecules can be tested.

A promising alternative toward achieving high throughput is nanopore sensing, a single-molecule technique that relies on applying an electric field to drive molecules through a nanosize opening, allowing one to measure changes in ionic currents relating to the size, shape, and charge of the molecule entering, or translocating, through the pore.^40^ Broadly speaking, there are two types of nanopores, biological, based on pore-like proteins embedded in membranes, and solid-state, which are fabricated by creating nanosized openings in a material. Platforms containing biological nanopores are commercially available from Oxford Nanopore Technology. However, due to size, these are mostly restricted to DNA sequencing^41^ or rely on protease cleavage of samples before nanopore measurements.^42^ Recently, the ability to discriminate between α-synuclein variants has been accomplished using biological nanopores.^43^ Using solid-state nanopores eliminates the need for fragmentation and allows the size of the nanopore to be directly tuned and optimized for the detection of the analyte of interest. Auspiciously for potential high-throughput applications, it has recently been demonstrated that solid-state nanopores could be manufactured at scale.^41^

Previously, solid-state nanopores have proven to be a useful tool for the detection of proteins,^44^ as well as a way to study their conformations and interactions.^45^ One of the major challenges associated with studying proteins in solid-state nanopores is the rapid speed at which they translocate. This challenge can be overcome with approaches such as employing bilayer-coated solid-state nanopores^46^ or by increasing the current bandwidth which increases the time resolution of the measurement.^47^ In one case, αS oligomerization was studied in solid-state nanopores using a Tween-20 coating.^48^ While these approaches are effective for studying single proteins, they are not easily adapted to multiplexed sensing. Current approaches are all based on observing single monomer or oligomer events, which can result in ambiguous signals. Discerning individual particle translocations can be challenging and is often based on observed differences in noise profiles (Figure 1D). Additionally, these methods have low throughput as multiplexing is not possible.

Since it has been demonstrated that the combination of solid-state nanopores and digitally encoded DNA nanostructures allows for highly multiplexed detection of single molecules,^49,50^ in this work, DNA nanostructures are used to study the effect of small-molecule inhibitors of αS secondary nucleation in a multiplexed assay. The advantage of this approach is that every oligomer in a particular sample has a distinctive ‘barcode’, that clearly identifies each individual particle, and allows aggregates from different inhibitor screens to be mixed together and tested simultaneously (Figure 1E). This enables the investigation of oligomer populations in more granular detail at higher throughput than was previously possible.

The small-molecule inhibitors tested in this work were determined via parallel work done by the authors.^39,51^ In the parallel work, inhibitors were initially identified via in silico docking to a putative catalytic site that promotes oligomer formation (Figure S1A) on the surface of αS fibrils followed by optimization in aggregation assays via active machine learning (Figure S1B).^39,51,52^ The application of nanopore detection to quantitative protein oligomer analysis therefore offers another useful application of this technique, with the potential of high-throughput analysis of a challenging target and an associated benefit to therapeutic programs targeting these misfolded protein aggregates.

## Results and Discussion

### DNA Nanostructure Design for the Capture of αS Oligomers

**Figure 2:**
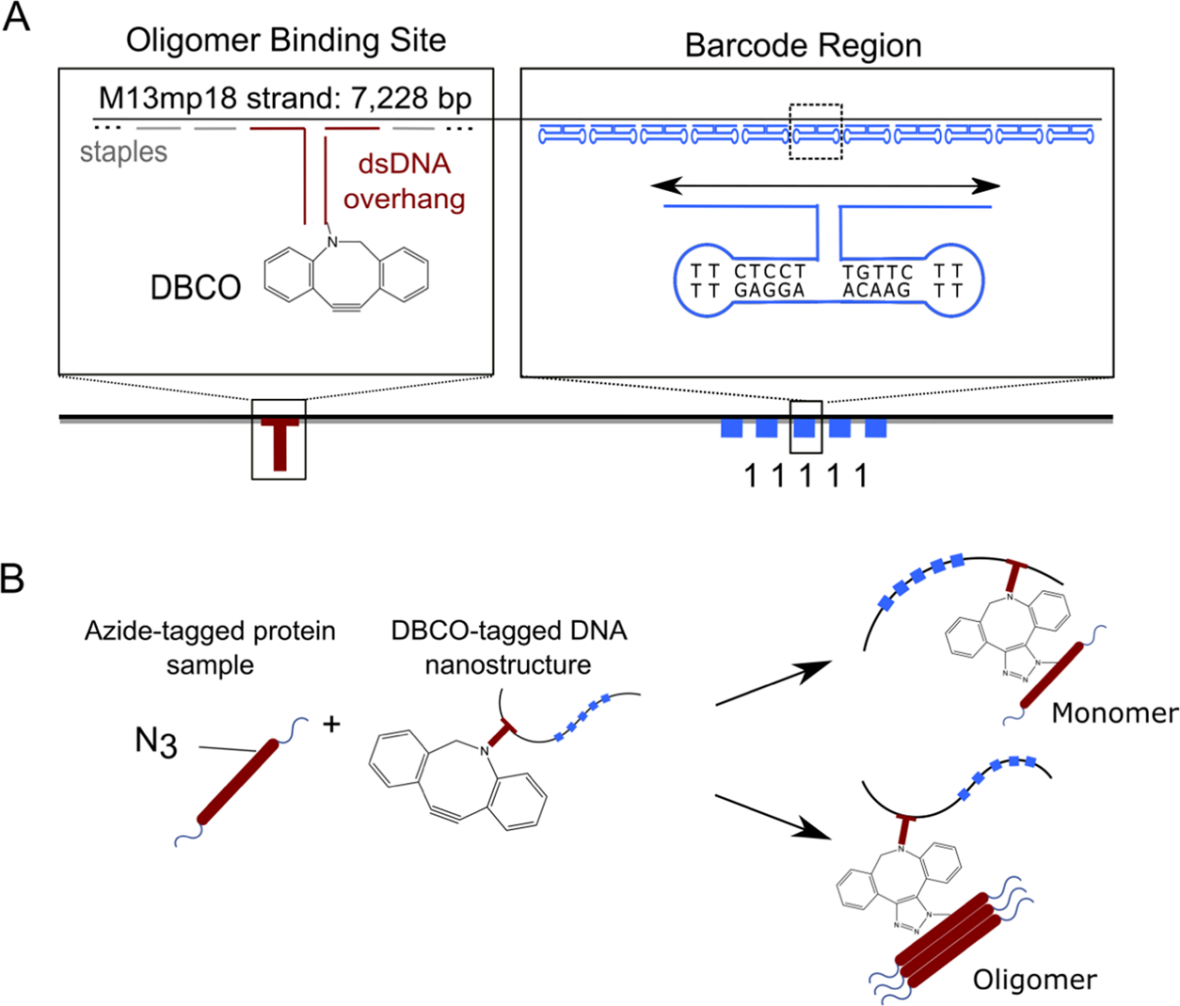
Design of a DBCO-DNA nanostructure for the capture of azide-labeled αS aggregates. (A) Schematic of the DNA nanostructure containing the DNA barcode region and a DBCO-tagged dsDNA overhang for click coupling to azide-tagged N122C-αS. DNA barcodes allow for a digital read-out of the single-molecule translocations using DNA dumbbells to create distinct 1 or 0 bits. (B) N122C-αS is tagged with iodoacetamide-PEG_3_-azide and then incubated with the DBCO-tagged nanostructure, allowing facile click coupling of the two components.

A DNA nanostructure was designed that could couple to azide-tagged αS aggregates and uniquely identify them (see Methods and Figure 2). Using a single-stranded DNA (ssDNA) as a scaffold, complementary staple DNA oligonucleotides were combined with additional oligonucleotides in a one-pot reaction and annealed. The additional oligonucleotides included DNA dumbbells which allowed for digitization of the structure. Their presence created a structured spike in the nanostructure, while their absence left a flat spacer region, corresponding to either a “1” or “0”. In the proof of concept presented here, only five spike/spacer regions were used, allowing for 2^5^ (32) combinations of barcodes. This design was based on previous work and was optimized to create clearly distinguishable spikes in nanopores of ∼15 nm diameter.^53^ However, this has the potential to be expanded with further optimization. We have previously shown one can fit 56 bits onto a single DNA carrier, allowing for a library of 2^56^ (>10^16^) molecules.^54^ Another section of the nanostructure contained two DNA strands, one 21 base pair (bp) sequence labeled with a dibenzocyclooctyne (DBCO) tag and one which had partial complementarity to both the scaffold and the sequence containing the DBCO, connecting the DBCO-tagged region to the rest of the nanostructure (Figure 2A).

The DBCO-labeled nanostructure was then combined with azide-tagged N122C αS samples for click coupling and subsequent detection (Figure 2B). The azide-tagged N122C αS monomer was prepared via reaction of reduced cysteine thiol with the iodoacetamide moiety of iodoacetamide-PEG_3_-azide. This reaction was monitored until completion via liquid chromatography–mass spectrometry (LCMS) (Figure S2). The monomer was isolated via SEC before use in aggregation experiments and subsequent coupling to the DNA tags.

### Detection of Stabilized αS Oligomers via DNA Nanostructures and Nanopores

We chose to first test the ability of the nanopores to act as a device to detect oligomers by using a stabilized oligomeric species. Stabilized αS oligomers have been extensively characterized previously.^55,56^ They are typically obtained using methods such as hyperconcentration and lyophilization and as such have limited physiological relevance. However, they do offer a useful test case for oligomer detection methods due to their greater stability, higher concentration, and larger size.^56^ Stabilized oligomers were used to optimize coupling times to the DBCO-tagged DNA barcodes and also to test whether an appreciable difference could be observed between monomeric and oligomeric samples in the nanopore. Successful click coupling of the samples was confirmed via PAGE (Figure S3, Table S1), where monomer-bound DNA was observable.

**Figure 3:**
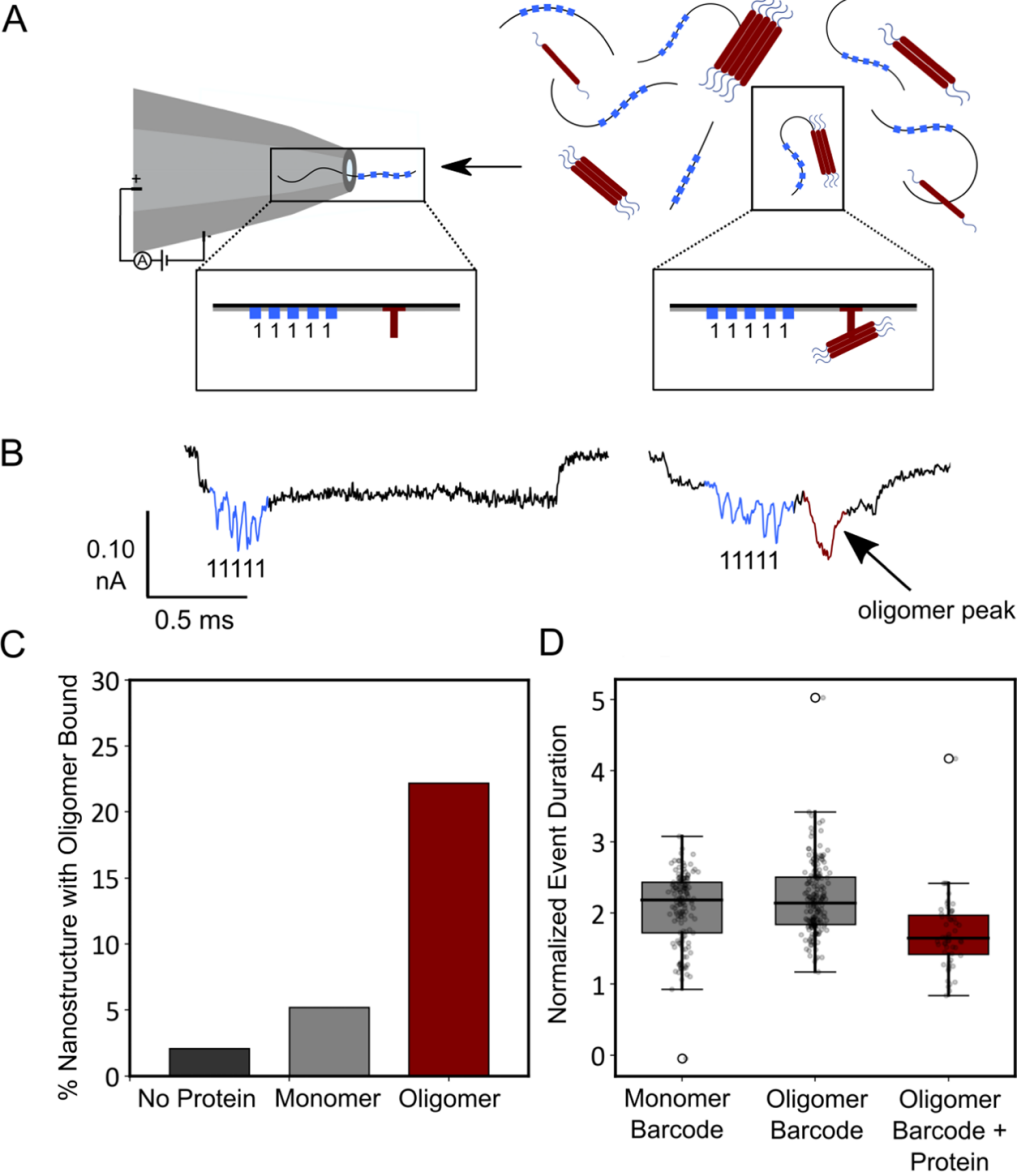
Detection of stabilized αS oligomers using nanopores. (A) Nanopore schematic representing the nanostructures with and without αS oligomers bound. (B) Current trace of the nanopore with no protein bound (left) and with an αS oligomer bound (right). (C) Percentage of events with spike for a control sample without αS added (*N* = 48), an αS monomer sample (*N* = 154), and an αS oligomer sample (*N* = 248). The samples with just αS monomers and stabilized αS oligomers act as negative and positive controls, respectively, and show a low percentage of false positives. (D) Normalized event duration (normalized to pore baseline current) for samples with barcode only (monomeric and oligomeric αS) or with a barcode and spike (oligomeric αS).

Samples of the coupled DNA–protein assemblies were pushed through a nanopore by using an electric current as a driving force (Figure 3A). The negatively charged nanostructure aided insertion into the pore when a current was applied. In this case, since the protein was also negatively charged at the pH used, the translocation was sped up. As the structures translocated through the nanopores, they created unique signals (Figure 3B). Monomer samples were compared with stabilized oligomer samples. Because the molecular weight of monomeric αS is ∼14 kDa, and as can be seen from the low percentage of additional spikes on the nanostructure from the monomer sample in Figure 3C, we can assume it is too small to be observed via the 15 nm nanopore. In this experiment, the samples containing no protein, only monomer, or stabilized oligomers were initially tested in different pores as a control to rule out any intersample interactions. The lack of events observed in the pure monomer sample allows us to clearly distinguish the samples with and without oligomers by their current traces and removes the monomers as a source of additional signal as their signal is too low to be detected in a nanopore of this diameter. This demonstrates how the customizable dimensions of solid-state nanopores can be utilized to focus on the subsample of interest. A significant difference in the percentage of events with proteins attached to the DNA barcodes was observed between the oligomeric and monomeric samples, demonstrating the potential utility of the approach for determining oligomer levels in a sample (Figure 3C).

It should be noted that the oligomeric samples also contained a significant proportion of monomer, which is otherwise challenging to separate entirely from the oligomer sample. Of the observed events in the oligomer sample, ∼22.2% had a protein oligomer spike attached to the DNA nanostructure. The rest of the events exhibited no spike due to being bound to a monomeric protein, which makes up the majority of the sample. The ability to measure with this background present is essential, given the additional time cost and potential bias introduced by the need to separate oligomeric species from the bulk monomer. These events can be separated both by observing the nanopore signal generated, where little to no protein spike signifies either an uncoupled DNA nanostructure or a nanostructure coupled to only monomer, as well as by using parameters such as event duration (Figure 3D). Because the protein is negatively charged, the event duration decreases in samples with bound proteins. As these samples were measured in different pores at different times, to ensure no cross-sample contamination and reliable controls, the duration must also be normalized to the baseline current (*I*_0_), and therefore a normalization was carried out as explained in the methods (eq 2).

### Effect of Inhibitor Molecules on αS Oligomer Production

Having optimized the conditions, we then tested more challenging “on time-course” samples. We carried out an aggregation beginning from monomer, under conditions designed to promote secondary nucleation.^21,57^ This assay has been fully characterized for an AlexaFluor-488 tagged N122C vs WT in previous works, and azide tagging did not substantially alter this behavior.^38,57,58^ Oligomer populations in this scenario are significantly lower in concentration compared to those in the stabilized oligomer case, and they are transient. On time-course samples of αS are only stable for ∼24 h post extraction, compared to αS stabilized oligomers which persist for up to a week after production if left at room temperature.

**Figure 4:**
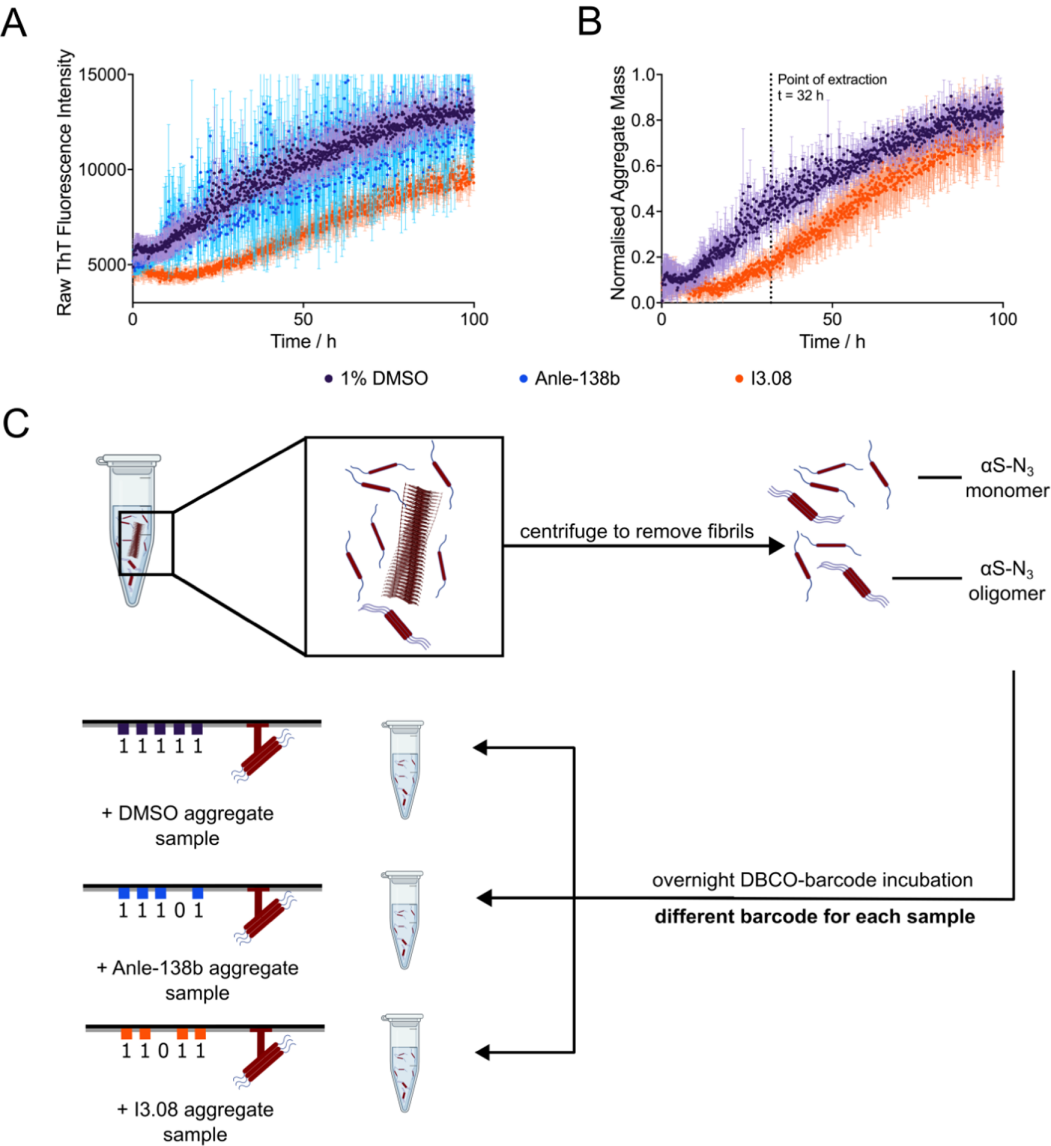
Preparation of an αS aggregation time-course in the absence and presence of inhibitor molecules, and extraction of oligomers. (A, B) Kinetic traces are shown of a 10 μM solution of azide-tagged N122C-αS supplemented with 100 nM preformed seeds (pH 7.4, 37 °C, shaking at 200 rpm, error bars denote SD) in the presence of 1% DMSO (purple), 25 μM Anle-138b (blue), or I3.08 (orange). The raw fluorescence (A) and normalized fluorescence (B) are shown. The end points were normalized to the αS monomer concentration at the end of the experiment, which was detected via the Pierce BCA Protein Assay at *t* = 100 h. Anle-138b could not be suitably normalized due to the noise of the sample. (C) Samples were extracted at 32 h from the time course of aggregation and centrifuged to remove fibrils from the mixture, leaving only αS monomers and soluble oligomeric species for analysis. These samples were then incubated with a unique DBCO-tagged DNA barcode overnight before analysis via solid-state nanopore detection.

The on-course experiment was designed to better mimic the processes and species that may occur in vivo. In order to induce αS aggregation via secondary nucleation in vitro at neutral pH, a small amount of preformed seed was added (100 nM monomer equivalents) in the presence or absence of aggregation inhibitors of interest (Figure 4A,B). The aggregation process was followed by using ThT fluorescence. The three samples of interest were a control containing only 1% dimethyl sulfoxide (DMSO), another control containing Anle-138b^24^ (an αS aggregation inhibitor that entered clinical trials) in 1% DMSO, and a small molecule identified previously via structure-based machine learning methods, I3.08, also in 1% DMSO. DMSO was used to dissolve the molecules before addition to the aqueous protein sample.

Our previous work reports that I3.08 binds to the fibrils, not the monomer or oligomers, and in so doing blocks autocatalytic aggregate formation.^39^ Fibrils are removed prior to nanopore measurement by centrifugation, so only the oligomer and monomer populations remain. The molecular mechanism of Anle-138b is not known in detail, aside from efficacy in aggregation assays. The aggregation was accelerated via shaking, which was necessary to complete the aggregation under cellular buffer conditions in an experimentally accessible time frame but created a more challenging paradigm for the inhibitors to function in. The inhibitors are capable of preventing aggregation only via secondary nucleation and not via fragmentation resulting from mechanical shearing. Nonetheless, a significant inhibition of fibril accumulation was still observed for inhibitor I3.08, although not for the control inhibitor Anle-138b. Samples were then taken midway through the time course to determine whether a reduction in oligomeric species was also observed.

Samples were extracted at 32 h into the aggregation time course and centrifuged to remove fibrils before click reaction of the azide-tagged αS with unique DBCO-tagged DNA barcodes overnight at a ratio of 1:1 (DBCO/initial monomer concentration) (Figure 4C). Each sample was labeled with a different DNA barcode: DMSO (11111), Anle-138b (11101), and I3.08 (11011). The aggregation reaction was diluted 2500-fold for this coupling, effectively quenching further aggregation. In the absence of conditions favoring phase separation,^59^ αS does not continue to aggregate under experimentally accessible timescales at concentrations below 5 μM regardless of the conditions.^25,60,61^ The DBCO/N122C-azide coupling required at least >3 h incubation time for the reaction to proceed significantly (Figure S4). The rate was tested by sampling 1, 3, and 12 h incubation times. No observable shift in PAGE was visible for 1 or 3 h, but an observable shift was visible for the sample incubated for 12 h. These results demonstrate that we can multiplex the samples without concern for significant further coupling reactions from any residual unreacted azide/DBCO species during the nanopore measurement. Concerns over the possible interchange of monomers in the sample between oligomers of different samples were addressed by the dilution at this stage with the expectation that interactions become essentially unfeasible. Additional repeats were done using duplexed DMSO and I3.08 samples (Figure S5). Similar results for samples tested in the duplex and triplex support this assumption. No separation of aggregate mixtures is carried out other than fibril removal, as this would drastically reduce throughput. Azide-tagged monomeric samples were obtained via SEC and incubated in a 1:1 ratio with DBCO-tagged DNA barcodes. Oligomeric samples resulting from aggregation reactions of azide-tagged monomers were similarly incubated with a 1:1 monomer equivalent ratio of DBCO-tagged DNA barcodes after fibril removal.

### Multiplexed Digital Nanopore Read-Out of the Effect of Inhibitor Molecules

**Figure 5:**
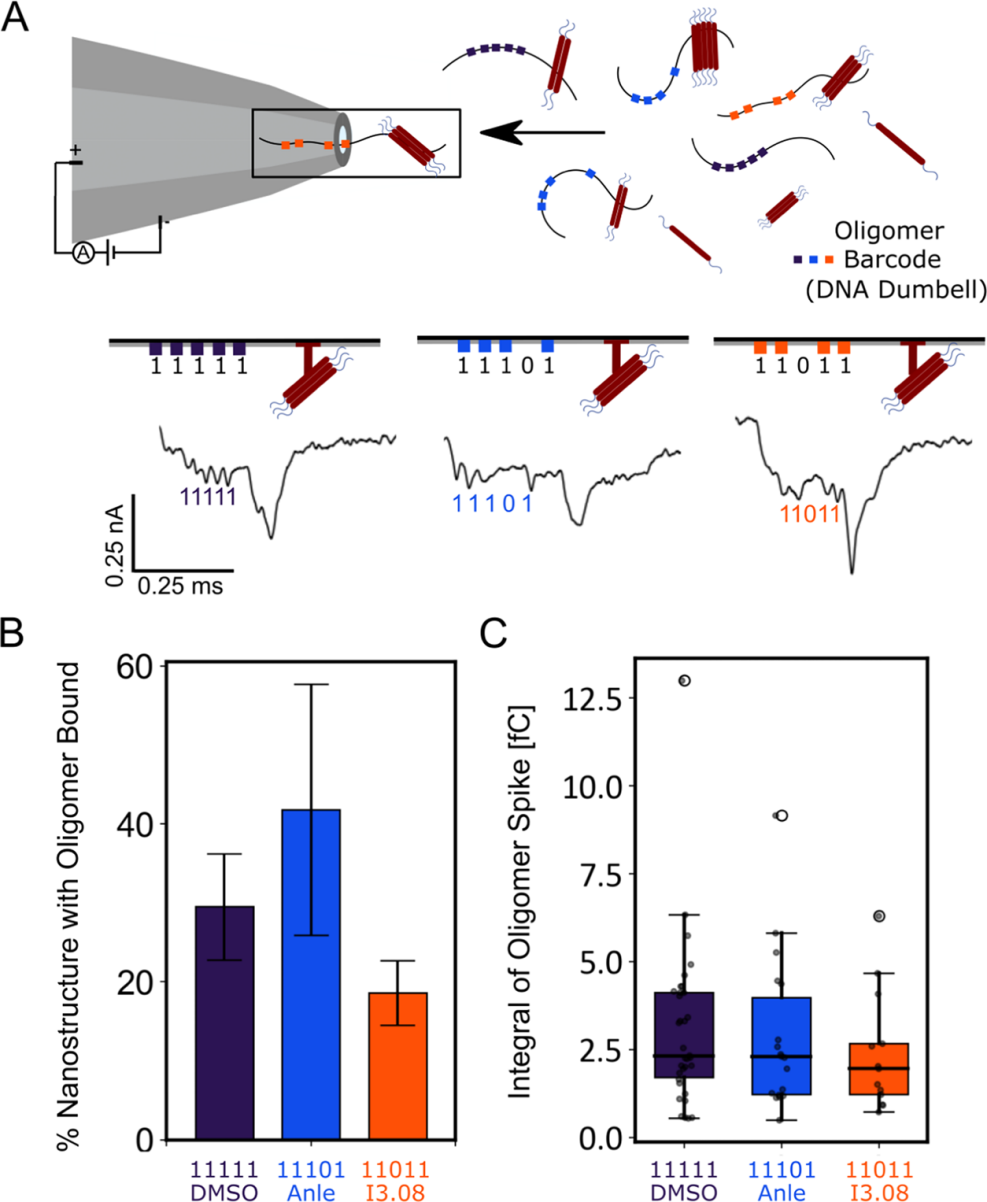
Schematic of the multiplexing pipeline and comparison of two different inhibitor molecules’ effects against time-course samples. (A) Samples are tagged with a unique DNA barcode that allows identification in a multiplexed mixture, increasing the throughput. The events observed as the oligomers translocate through the nanopore can then be analyzed to give an oligomer number per tag, and a relative area under the curve of each tag, proportional to oligomer size. (B) Fraction of events with an oligomer bound to the DNA barcode: DMSO (purple) (*N* = 114 ± 7), Anle-138b (blue) (*N* = 43 ± 16), and I3.08 (orange) (*N* = 90 ± 4). The standard deviation comes from repeats where the samples were combined, diluted in measurement buffer, and measured for ∼1 h. (C) Area of the current drop of the protein spike caused by bound oligomer in the DMSO (purple), Anle-138b (blue), and I3.08 (orange) samples. A larger area implies that larger species are bound to the barcode on average.

Using the method described above, the samples were then injected into the nanopore. Analysis of both the number of events containing a discernible DNA barcode and an attached protein spike, and the area of the protein spike, showed a change in oligomer distribution compared to the DMSO control (Figure 5). The DNA barcode is the observable quantity, and so a ratio of the barcode with bound oligomer versus unbound was calculated as described in the Methods section. The nanopores were fabricated to be 12–15 nm such that monomeric proteins would not be observable, while oligomeric species would be observable. The DMSO sample barcode was 29.8% bound to protein oligomers, the Anle-138b sample barcode was 41.8% bound, and the I3.08 sample barcode was 14.4% bound (Figure 5B). The size distribution of the oligomers broadly matched this trend, showing decreasing oligomer mass from the DMSO sample to the Anle-138b sample, which contained a large number of small oligomers as explained below, and lastly the I3.08 sample (Figure 5C). This was calculated using eq 3. As the samples were run simultaneously in the same pore, no normalization was required. These results show that compound I3.08 reduced oligomer production relative to the untreated control and that it was a better inhibitor of oligomer production than Anle-138b.

Interestingly, first I3.08 and DMSO were tested in duplex, and a similar baseline level noise (∼6 pA) was maintained throughout the measurement. With the addition of Anle-138b in triplex with the other samples, the noise level increased (Figure S6). This was consistent with the kinetic data (Figure 4A). The Anle-138b sample exhibited a noisy kinetic trace, consistent with increased formation of particulates, and had a correspondingly greater oligomer population.This may have resulted in part from Anle-138b’s reportedly low solubility.^24^ The increase in nanopore noise is most likely due to the larger oligomers present rapidly translocating through the pore at the beginning of the measurement. After 3 min, most of the larger oligomers have translocated through the pore, which leads to the baseline current and noise resuming back to normal. This is also consistent with the number of events measured for Anle-138b (*N* = 43), where fewer discernible events with Anle-138b barcode 11101, as compared to DMSO barcode 11111 (*N* = 114) and I3.08 barcode 11011 (*N* = 90), were observed despite all samples being added at equal concentration.

### Comparison with a Micro Free-Flow Electrophoresis (μFFE) Method

For comparison, a state-of-the-art technique in protein oligomer detection is micro free-flow electrophoresis (μFFE), which allows full characterization of the oligomer distribution in physiological conditions, and has been previously applied to ascertaining oligomer populations in the presence of a closely structurally related inhibitor to the one used here.^38,39^ While μFFE requires insoluble fibrils to be removed via centrifugation, no further separation is required, as the technique separates the monomeric fraction from the oligomeric fraction in situ using an electric field across the particle stream that deflects particles based on their electrophoretic mobility. The only disadvantage is the relatively low throughput. In that work, a molecule (I3.02) induced a 37% delay in the half-time of aggregation compared to the negative 1% DMSO control. As a result, there was a 75% reduction in the mass of oligomers present at half of the time of the negative control. The aggregation kinetics were carried out under similar conditions as used here, with the primary difference in that work being the higher concentration of αS monomer and the molecule (100 μM αS, 50 μM molecule). In this work, molecule I3.08 induced a 57% delay in relative half-time, and as measured by nanopore detection, the drop in oligomer events observed was 48% and the drop in oligomer mass was 22% compared to the negative DMSO control. Anle-138b was shown to have lower effectiveness in terms of oligomer number reduction and oligomer mass reduction via both techniques, so the ranking of effectiveness between nanopore detection and μFFE is in agreement.

The strategy here was to create a novel screening approach for aggregation inhibitors and not to fully characterize the aggregation time course, though this would represent a valid application of the technology. This has however been done multiple times previously^21,57,58^ while oligomer inhibitory screening assays are scarcer, due to difficulty in applying existing methodologies with low throughput. The comparison between μFFE, one of the methods used to carry out a full-time course characterization^38,58^ and then to characterize inhibitor potency,^39^ and the nanopore method shown here, demonstrates that both are effective at ranking molecules in terms of molecule potency.

### Discussion

We have reported a nanopore detection method for misfolded protein oligomer detection and analysis, with a detection limit on par with current state-of-the-art techniques but with significantly greater potential for throughput. To illustrate the method, we applied it to detect the inhibition of αS oligomer production by small molecules in clinical development. This result was obtained with the additional benefit of multiplex capability and higher throughput.

While the nanopore system has many advantages, there are also some drawbacks. A drawback of the dilution step required for measurement in the nanopore is the possibility that some of the oligomers may dissociate during the DBCO coupling step (12 h) due to the large dilution (2500-fold). This is a feature of most single-molecule techniques that require low concentrations in order to have a clear signal-to-noise ratio. However, αS is a useful test case in this scenario given that its kinetics are relatively slow and its oligomers are stable^38,62^ over the time scales investigated so we consider the measured sample to be a reasonable reflection of the population present at the extraction stage. In further developments, a cross-linking step could be introduced to ensure that the extracted protein sample exactly matches the one measured. This carries the risk of cross-linking separate oligomers (potentially mitigated by appropriate dilution) and adds further processing steps, issues that we sought to avoid in the interests of throughput and preventing biasing of the oligomer population. Alternatively, if the dissociation rate in a particular case was a cause for concern, a more reactive click pair could be employed than the one used here or the coupling could be carried out at a higher concentration (followed by dilution immediately prior to measurement) to obtain coupling over a shorter time scale and slow dissociation. A restraint on the click coupling reaction is that the sample conditions cannot be altered in terms of pH or temperature, as this would affect the oligomer distribution.

An additional concern with the nanopore measurement is the high salt concentration required for the measurement, which may perturb the aggregate distribution. However, the click chemistry reaction was performed in phosphate-buffered saline (PBS), and the samples were mixed only in the detection buffer directly before measurement. The ratio of protein bound to unbound DNA nanostructures also did not change over the time of the observation (Figure S7) suggesting this is not a major issue. Again, cross-linking could remove this problem if necessary. In the interests of throughput, however, and for cases where there is a clinical trial benchmark, all that would be required is a relative measurement to compare the effect of different inhibitors. As the samples are measured under the same conditions, a ranking of effectiveness could still be obtained. For protein systems that aggregate very rapidly, the concern is more that the monomers and oligomers may further aggregate during the click reaction rather than dissociate. We anticipate that for almost all proteins, the significant dilution should quench aggregation to a rate that is negligible over the time span of the coupling reaction.

Finally, using nanopores as a tool to measure oligomers does have a fundamental size limit in that particles larger than the diameter of the pore and smaller than the resolution limit will not be detected. However, with a degree of prior knowledge, the nanopore diameter can be appropriately tailored to the size distribution of interest, allowing the sampling of a representative portion of the population.

## Conclusions

The results that we have presented illustrate an approach for investigating protein assemblies that are both transient and present at very low concentrations. We have applied this method to the scenario of early drug discovery for Parkinson’s disease and synucleinopathies in general, where misfolded αS oligomers are considered to be key to pathology. We also show comparable performance to existing single-molecule techniques but with greater potential for throughput due to the ability to multiplex and upscale. With the introduction of artificial amino acids bearing azides into in vivo models of disease,^63^ this also represents a potential approach for directly quantifying oligomer populations in such models, utilizing the biorthogonality of the click reaction employed here. We anticipate that this approach could be of significant benefit to researchers working in the field of protein misfolding diseases and protein multimerization and in early-stage drug discovery research in general.

## Materials and Methods

### Compounds and Chemicals

Compounds were purchased from MolPort (Riga, Latvia) or Mcule (Budapest, Hungary) and prepared in DMSO to a stock of 5 mM. All chemicals used were purchased at the highest purity available.

### Recombinant αS Expression

Recombinant αS was purified based on previously described methods.^60,61,64^ The plasmid pT7–7 encoding human αS was transformed into BL21 (DE3) competent cells. Following transformation, the competent cells were grown in 6 L of 2xYT media in the presence of ampicillin (100 μg/mL). Cells were induced with IPTG, grown overnight at 28 °C and then harvested by centrifugation in a Beckman Avanti JXN-26 centrifuge with a JLA-8.1000 rotor at 6240*g* (Beckman Coulter, Fullerton, CA). The cell pellet was resuspended in 10 mM Tris, pH 8.0, 1 mM ethylenediamine tetraacetic acid (EDTA), and 1 mM phenylmethylsulfonyl fluoride (PMSF) and lysed by sonication. The cell suspension was boiled for 20 min at 85 °C and centrifuged at 39,000*g* with a JA-25.5 rotor (Beckman Coulter). Streptomycin sulfate was added to the supernatant to a final concentration of 10 mg/mL and the mixture was stirred for 15 min at 4 °C. After centrifugation at 39,000*g*, the supernatant was taken with an addition of 0.36 g/mL ammonium sulfate. The solution was stirred for 30 min at 4 °C and centrifuged again at 39,000*g*. The pellet was resuspended in 25 mM Tris, pH 7.7, and the suspension was dialyzed overnight in the same buffer. Ion-exchange chromatography was then performed using a Q Sepharose HP column of buffer A (25 mM Tris, pH 7.7) and buffer B (25 mM Tris, pH 7.7, and 1.5 M NaCl). The fractions containing αS were loaded onto a HiLoad 26/600 Superdex 75 pg Size Exclusion Chromatography column, and the protein (≈60 mL @ 200 μM) was eluted into the required buffer. The protein concentration was determined spectrophotometrically using ε280 = 5600 M^–1^ cm^–1^. The cysteine-containing variant (N122C) of αS was purified by the same protocol with the addition of 3 mM DTT to all buffers.

### Azide Labeling of αS

αS N122C protein was azide-labeled to enable click coupling to DNA tags. N122C (200 μM, PBS, pH 7.4) was incubated with TCEP-HCl (5 equiv) for 1 h at room temperature (RT). The reduced N122C was then desalted with a 5 mL HiTrap desalting column (Cytiva, 29-0486-84), eluted in PBS, pH 7.4, 10 mM EDTA, and kept on ice. The extent of the reduction was then established via Ellman’s method, and a sample was taken for LCMS analysis. The protein was then incubated with iodoacetamide-PEG3-azide (10 equiv) for 3 h at RT, and samples were taken subjected to QTOF MS/MS analysis with a VION mass spectrometer to ascertain the progress of the reaction (Figure S2). Deconvolution was conducted with UNIFI software. Upon reaction completion, the reaction mixture was separated on a Superdex 75 10/300 GL column (GE Healthcare) at a flow rate of 0.5 mL/min and eluted in PBS buffer to isolate the monomeric fraction and buffer exchange into PBS. The protein concentration was determined spectrophotometrically using ε280 = 5600 M^–1^ cm^–1^.

### αS Seed Fibril Preparation

αS fibril seeds were produced as described previously.^61,64^ Samples of αS (700 μM) were incubated in 20 mM phosphate buffer (pH 6.5) for 72 h at 40 °C and stirred at 1500 rpm with a Teflon bar on an RCT Basic Heat Plate (IKA, Staufen, Germany). Fibrils were then diluted to 200 μM, aliquoted, flash frozen in liquid N_2_, and finally stored at −80 °C. For the use of kinetic experiments, the 200 μM fibril stock was thawed and sonicated for 15 s using a tip sonicator (Bandelin, Sonopuls HD 2070, Berlin, Germany), using 10% maximum power and a 50% cycle.

### αS Stabilized Oligomer Preparation and Subsequent Click Coupling

αS stabilized oligomers were produced as described previously.^56^ Monomeric αS was dialyzed into distilled water overnight at 4 °C, using 3.5 kDa MWCO dialysis membranes. Six mg of the dialyzed protein was aliquoted into 15 mL tubes, flash frozen in liquid nitrogen, and lyophilized for ca. 48 h at room temperature. To prepare the oligomeric samples, the 6 mg of protein was resuspended in a total of 500 μL of PBS to obtain a final protein concentration of ca. 800 μM. The solution was centrifuged if necessary (1 min, 1000*g*) to get rid of bubbles formed during the resuspension process. The protein solution was filtered through a 0.22 μm syringe filter and incubated in 1.5 mL tubes at 37 °C for 20–24 h under quiescent conditions. The resultant protein solution was ultracentrifuged (1 h, 288,000*g*) to remove any fibrillar species that may have formed during the incubation period, and the supernatant was removed and retained. Each aliquot of supernatant was passed through four 0.5 mL 100 kDa centrifugation filters sequentially (2 min, 9300*g*) in order to remove excess monomeric protein as well as the low levels of very small oligomers. To estimate the total mass concentration of the final oligomeric solution (i.e., total concentration in monomer equivalents), the absorbance was measured at 275 nm, using a molar extinction coefficient of 5600 M^–1^ cm^–1^. This preparation results in an overall oligomeric yield of ca. 1%. Samples were then diluted to a final concentration of 88 nM monomer equivalents in PBS and incubated overnight with a final concentration of 4 nM of DBCO-tagged DNA nanostructure. The reason this excess was used was to attempt to ensure 1 DBCO tag per oligomer and prevent over-tagging (each stabilized oligomer has a reported average monomer count of 22^56^). Subsequent on time-course experiments were carried out with 1:1 labeling of the DBCO/monomer given the large excess of monomer:oligomer expected in these samples.

### DBCO-DNA Nanostructures

DNA constructs with different barcoded regions plus a DBCO-labeled overhang sequence were created. Each DNA construct was synthesized by pairing a linearized 7.2 kbp single-stranded (ss) M13mp18 DNA with 40 nucleotide staples complementary to the scaffold in order to create a full linearized dsDNA. The scaffold and staples are annealed for 45 min in a thermocycler. Using a 100 kDa Amicon filter, the sample is then filtered and stored in 10 mM Tris 0.5 mM MgCl_2_ pH 8. The concentration is then measured in a nanodrop spectrophotometer with typical yield of DNA nanostructure ranging from 75 to 95%. The barcoded region design follows a previous work with dumbbells optimized for read-out in 15 nm nanopores.^65^ Each “1” bit is made of 11 simple dumbbell hairpin motifs to create the structural spikes that act as a barcode on the DNA nanostructure. This can be optimized to have fewer dumbbells per spike, if needed. The exact sequences with their numbers are shown in Table S2 in the Supporting Information following a previous work.^65^ The overhang was created by replacing oligo No. 142 with 61 bp segment containing 40 bp to match the scaffold and a 21 bp oligo complementary to another DNA sequence containing a DBCO label. The 21 bp dsDNA overhang is not large enough to generate a current blockade (an observable signal in the nanopore), which has been confirmed by observation. These sequences can be found in Supporting Information Table S3.

### Aggregation Kinetics and Subsequent Click Coupling

Azide-labeled αS N122C (10 μM) was supplemented with seed (100 nM) under shaking (200 rpm) at 37 °C, PBS pH 7.4, and either 1% DMSO or 25 μM molecule in 1% DMSO. Samples were extracted at the *t*_1/2_ of the DMSO sample (30 h). Fibrils were removed by centrifugation (21130*g*, 10 min, 25 °C). Samples were then diluted to 4 nM monomer equivalents in PBS and incubated overnight with 1 equiv (relative to initial monomer concentration) of DBCO-tagged DNA nanostructure.

### Nanopore Fabrication and Measurement

The nanopores are made of commercially available quartz capillaries (0.2 mm ID/0.5 mm OD Sutter Instruments, CA). A laser-assisted pipet puller (P-2000, Sutter Instrument, CA) is used to create nanopores with diameters of 10–15 nm. Sixteen conical nanopores are then placed in a custom-templated polydimethylsiloxane (PDMS) chip containing a communal cis reservoir and individual trans reservoirs. In order to generate the current, silver/silver chloride (Ag/AgCl) electrodes are connected to the cis and trans reservoirs in the PDMS chip. In the baseline buffer solution for the stabilized oligomers (4 M LiCl, 1X TE, pH 8.0) and the on-pathway samples (2 M LiCl, 1X TE, pH 8.0), a current–voltage curve is taken in order to estimate the nanopore size. Only one nanopore is measured at a time due to the electronics; thus, the trans reservoir contains the electrode with a 500 mV bias voltage, and the central cis reservoir which contains the sample is grounded. The measurement is then run for 1–2 h until 1500–3000 events are gathered. Typically, of these events, 30% are unfolded and are then analyzed.

Current signals are collected using an Axopatch 200B patch-clamp amplifier (Molecular Devices, CA). The setup is operated in whole-cell mode with the internal filter set to 100 kHz. An 8-pole analogue low-pass Bessel filter (900CT, Frequency Devices, IL) with a cutoff frequency of 50 kHz is used to reduce noise. The applied voltage is controlled through an I/O analog-to-digital converter (DAQ-cards, PCIe-6251, National Instruments, TX). A LabView program records the current signal at a bandwidth of 1 MHz.

### Nanopore Data Analysis

The experimental data files are stored as technical data management streaming (TDMS) files from the Labview program, recording the raw traces. First, a translocation finder python script is used that identifies the events from the raw traces using user-defined thresholds (minimum 0.3 ms duration, minimum 0.1 nA current drop) and stores them in an hdf5 file. This can be found at https://gitlab.com/keyserlab/nanopyre. Next, the hdf5 file is loaded into the GUI categorizer python script, found here: https://gitlab.com/keyserlab/nanopycker. Using this, the events are printed, and the user can manually sort the events time efficiently into different categories and later print events from the hdf5 file that are assigned to a specific category. In this case, the categories were barcode without protein and barcode with protein. The percentage of events with oligomer bound is then calculated using
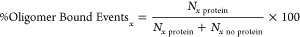
1where *x* is the barcode. This is used in Figures 3C and 5B. The duration of the events in Figure 3D is calculated using
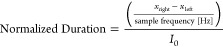
2where *x*_right_ is the position of the end of the event and *x*_left_ is the position of the end of the event. The sampling frequency is 1,000,000 Hz. *I*_0_ is the baseline current because different pores were used for different measurements with different baselines.

The GUI categorizer is used again on the events with protein to calculate the ECD of the protein spike using
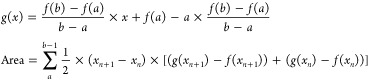
3where *f*(*x*) is the current at point *x*; *a* and *b* are the left and right bounds of the region of interest, respectively; and *g*(*x*) is the equation of the line connecting *a* and *b*.

### Mass Spectrometry

10 μM preformed αS was incubated with 25 μM molecule in 20 mM sodium phosphate buffer (pH 4.8) supplemented with 1 mM EDTA overnight under quiescent conditions at room temperature. The supernatant was removed for analysis by using a Waters Xevo G2-S QTOF spectrometer (Waters Corporation, MA).

## Supporting Information Available

Gel analysis of the azide-DBCO reaction between tagged protein and tagged DNA, LCMS analysis of the azide tagging of N122C, comparisons of nanopore measurements run in duplex and triplex, comparisons of noisy and clear samples, and monitoring of protein binding level for the duration of the experiment and sequences for all DNA used (PDF)
