# Supplementary material for: Multiplexed Digital Characterization of Misfolded Protein Oligomers via Solid-State Nanopores
Source: J Am Chem Soc. 2023 Nov 16;145(47):25776–88. doi: 10.1021/jacs.3c09335 (PMC10690769; doi:10.1021/jacs.3c09335)
Supplement: Supplementary file 1 — ja3c09335_si_001.pdf [file ja3c09335_si_001.pdf]

Multiplexed Digital Characterization of Misfolded Protein Oligomers via Solid-State Nanopores

Sarah E. Sandler[a#], Robert I. Horne[b#], Sara Rocchetti[a], Robert Novak[a], Nai-Shu Hsu[b], Marta Castellana Cruz[b], Z. Faidon Brotzakis[b], Rebecca C. Gregory[b], Sean Chia[b,c], Gonçalo J. L. Bernardes[b], Ulrich F. Keyser[a] and Michele Vendruscolo[b*]*

[a]Cavendish Laboratory, Maxwell Centre, Department of Physics, University of Cambridge, Cambridge, CB3 0HE, UK.

[b]Centre for Misfolding Diseases, Yusuf Hamied Department of Chemistry, University of Cambridge, Cambridge, CB2 1EW, UK.

[c]Bioprocessing Technology Institute, Agency for Science, Technology and Research (A*STAR), Singapore, 138668.

#Authors contributed equally to this manuscript

Email: *mv245@cam.ac.uk, *ufk20@cam.ac.uk

Keywords

Parkinson's disease; α-synuclein; protein aggregation; structure-based iterative ML; drug discovery; single molecule analysis; nanopore detection; multiplexed HTS

Methods

PAGE gel

Polyacrylamide gels (10% v/v, with 0.5X, pH 8, Tris-Borate-EDTA and 11 mM $MgCl_2$) were hand-cast on a PAGE loading gel setup. Details on the PAGE gels recipes can be found in **Table 1**. Once all the gel mixture additions (**Table 1**) were mixed together, 1% (w/v) APS and 0.07% (w/v) TEMED were added and the mixture was immediately vortexed and poured in between two PAGE glass slides using a Pasteur pipette. The gel comb was promptly inserted and the gel was left to polymerise for at least 45 minutes and a maximum of 1 hour. Gels were run for 120 minutes at 100 V in a running buffer containing 0.5x TBE and 11 mM $MgCl_2$. DNA was stained using GelRed® (Biotium) for 15 minutes under constant shaking. Imaging was performed using a Gel-DocIt imaging system by UPV using Visionworks software under UV excitation light and exposure times varying from 5 to 10 seconds. To check for the binding of the copper-free click

chemistry reaction between DBCO-DNA and azide-labelled αS and secondarily to optimize the reaction conditions, DBCO-DNA was incubated in a 1:1 ratio (monomer : DBCO) for 1h, 3h, and overnight **(Figure S3)**.

**Gel mixture addition**	**Amount [mL]**	**Final concentration**
Acrylamide/bis-acrylamide, 30 % solution	5	10 %
10x Tris-Borate-EDTA (TBE)	0.75	0.5x
0.5 M $MgCl_2$	0.33	11 mM $MgCl_2$
MilliQ water	8.92	
Total volume	15	
**Polymerisation initiatiors**		
10 % (w/v) ammonium persulfate (APS)	0.15	0.1 %
N,N,N',N'-Tetramethylethylendiamine (TEMED)	0.01	0.07 %

Table S1: Recipe for a 10% PAGE gel.

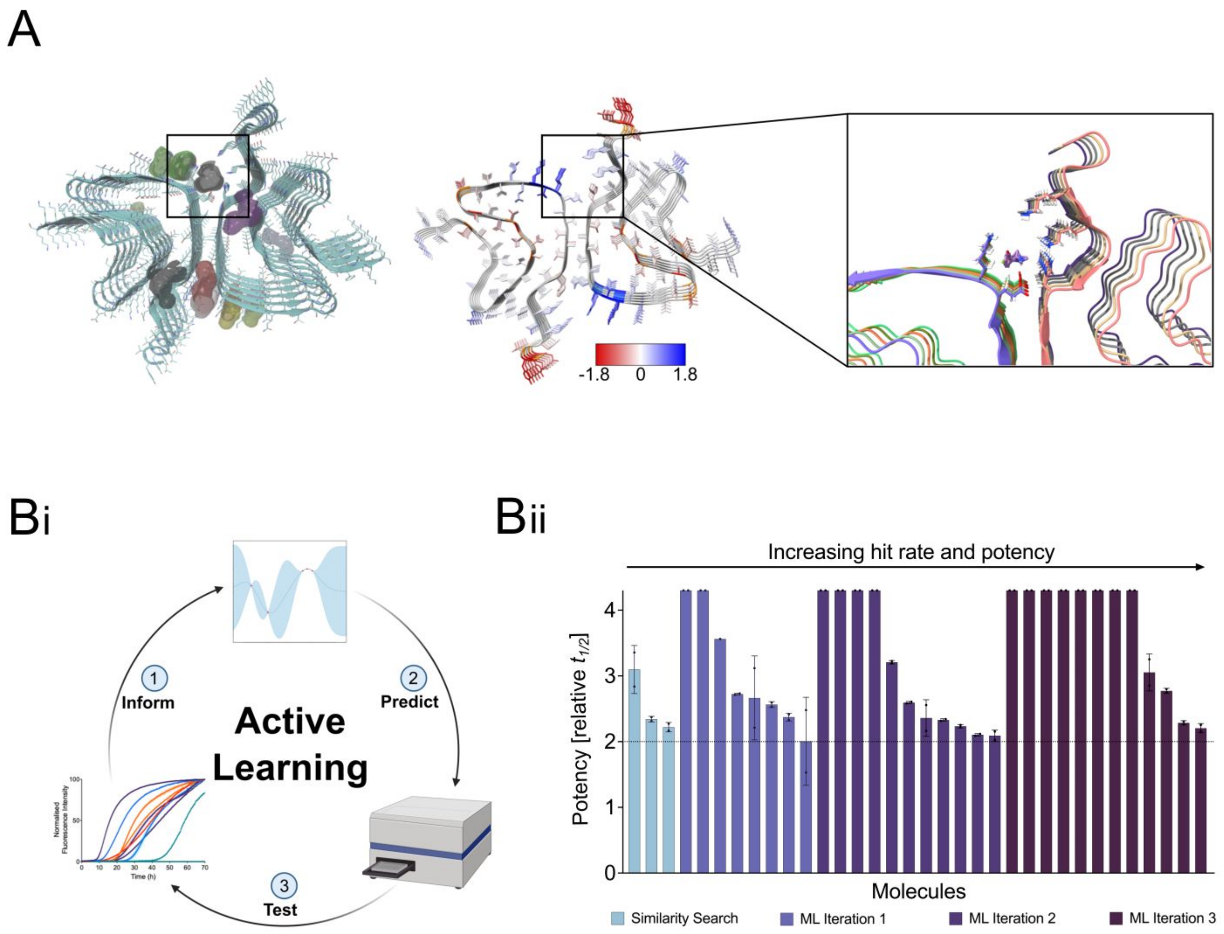

Figure S1. (A) A structure-based iterative machine learning strategy comprised of docking simulations followed by cycles of active machine learning was employed to identify secondary nucleation inhibitors. The former consisted of docking small molecules to the catalytic aggregation sites on the fibril surface, identified in the cryo-EM structures of the fibrils via pocket identification software[1], surface solubility predictions (colour coded from low to high, red to blue)[2] and experimental knowledge. **(Bi)** Iterative active machine learning was applied to a set of experimentally validated hits, initially identified via docking, and their analogues. **(Bii)** This successively improved molecule potency with every iterative experimental screen, as the information from each screen was fed back into the active learning algorithm. The 8th hit from

iteration 3, I3.08, is used as a tool compound here. Adapted with permission from Robert I. Horne[3]. Copyright 2023 *bioRxiv*.

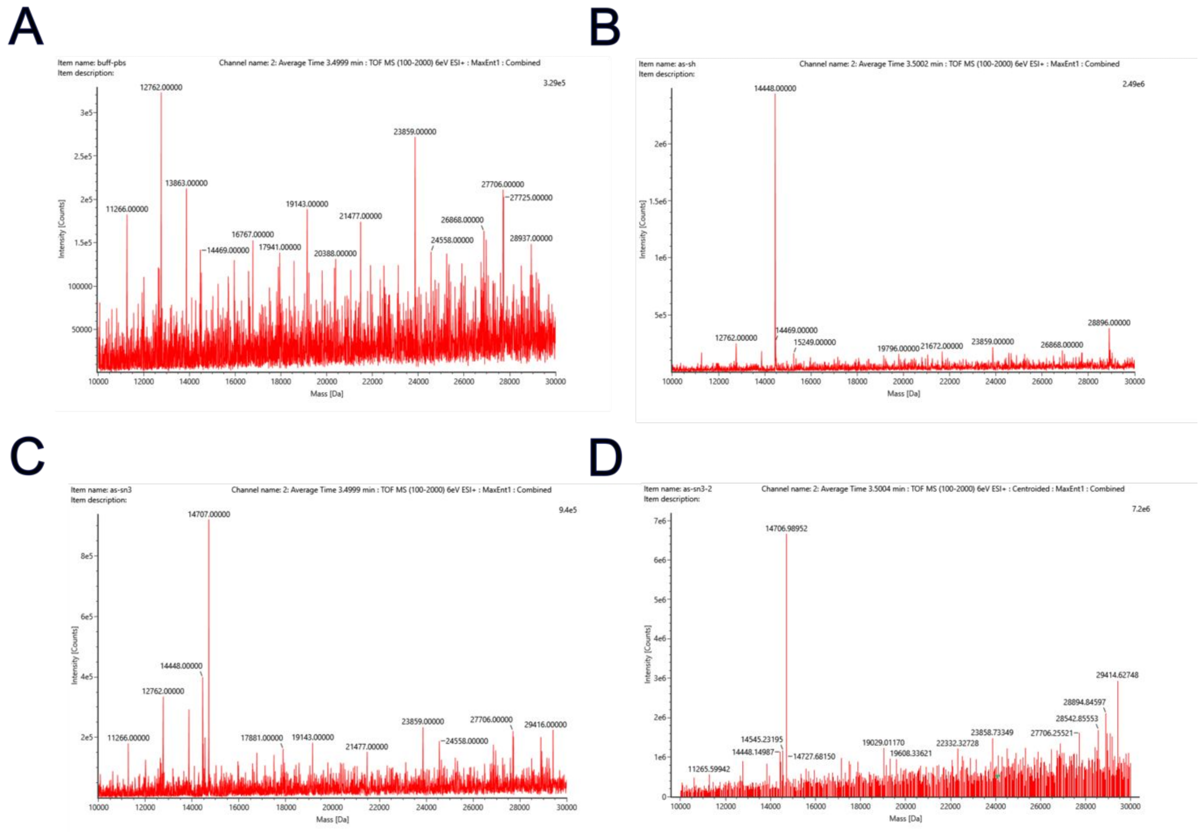

Figure S2: LC-MS data for the reaction progress of the azide linking step to N122C-αS. (A) PBS buffer control. (**B**) N122C (1 μM, PBS buffer) after reduction with TCEP to remove dimers, showing a single peak at 14448 Da. (**C**) Reduced N122C (1 μM, PBS) after a 2 h incubation with iodoacetamide-PEG$_3$-azide. The labelled peak, at 14707 Da, is prominent but residual unlabelled N122C remains. (**D**) After 3 h almost all of the monomer has been labelled.

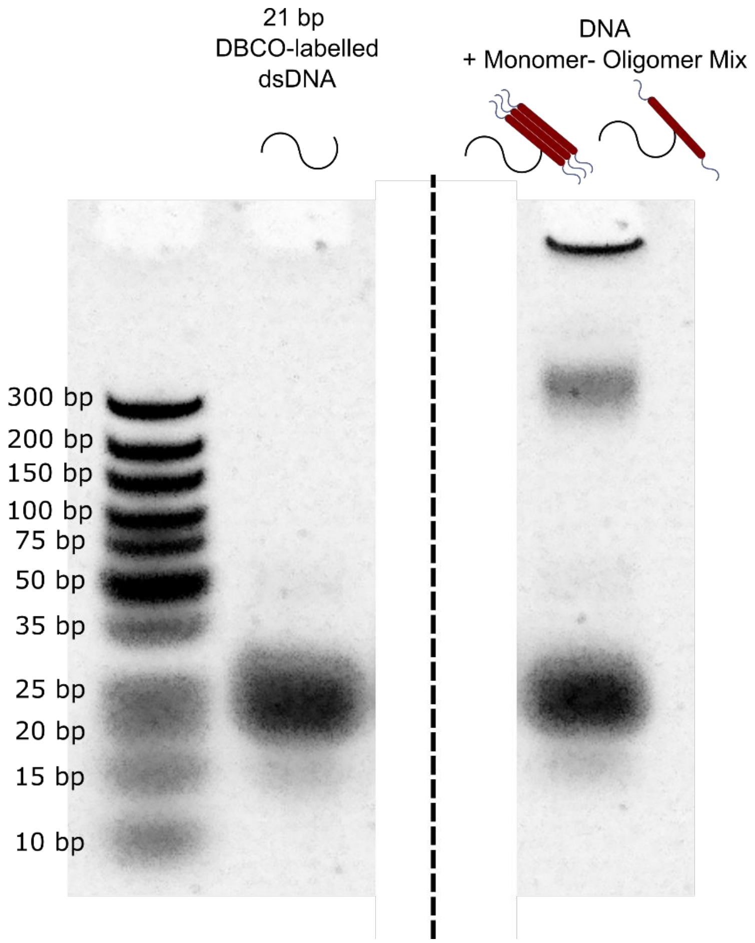

Figure S3. Monomer-bound DNA observed using PAGE. Column 1 shows 21 bp dsDNA and Column 2 shows a mixture of 21 bp dsDNA mixed with a partially converted oligomer and monomer sample. The monomer is 14 kDA (10 kDA ~ 270 bp), which matches the strongly stained band when added to the 21 bp DNA. The DNA retained in the well may be due to aggregates formed in the sample that cannot enter the gel.

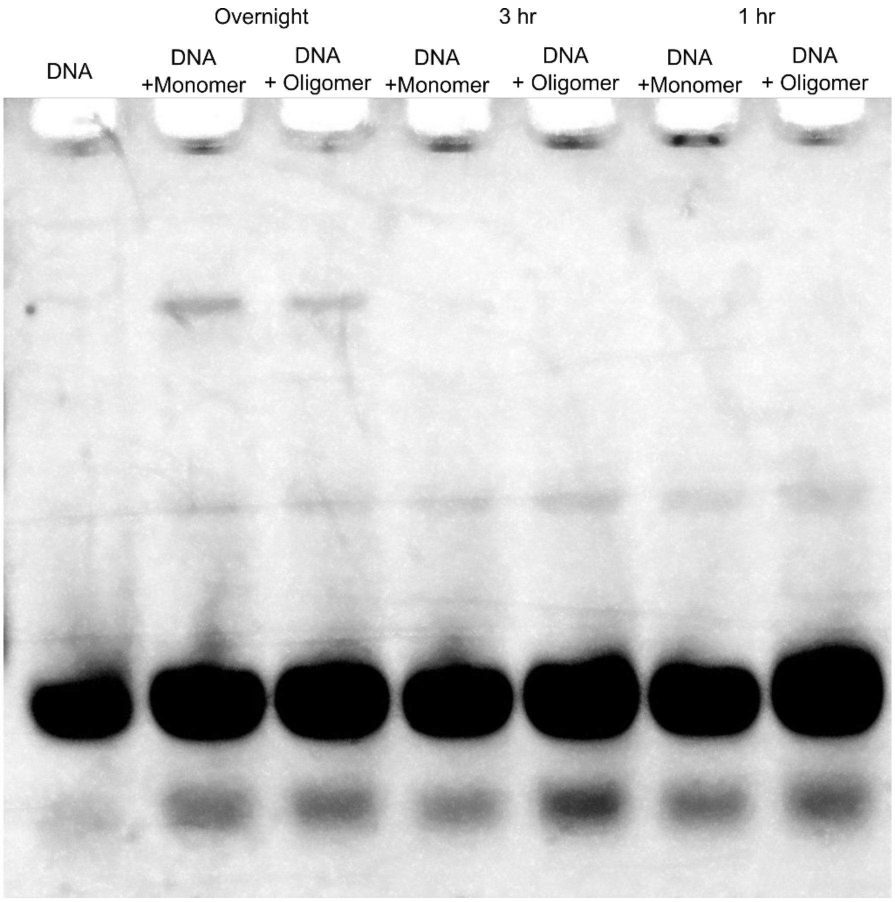

Figure S4. DBCO-azide click reaction as observed via PAGE. Monomeric αS (5 nM) and oligomeric αS taken from the half time of an aggregation reaction (5 nM) were incubated with 21 bp dsDNA linked to DBCO (5 nM) as a test of the required reaction time. Bound monomeric αS is only observed in the overnight incubation but not for the 3 h and 1 h incubation times. Oligomeric αS is not observed in any lane as the concentrations are too low and the oligomers may not be stable in the gel. The bands from bottom to top have been identified as: ssDNA, dsDNA, a contaminant from the DNA synthesis, and monomeric αS bound to dsDNA.

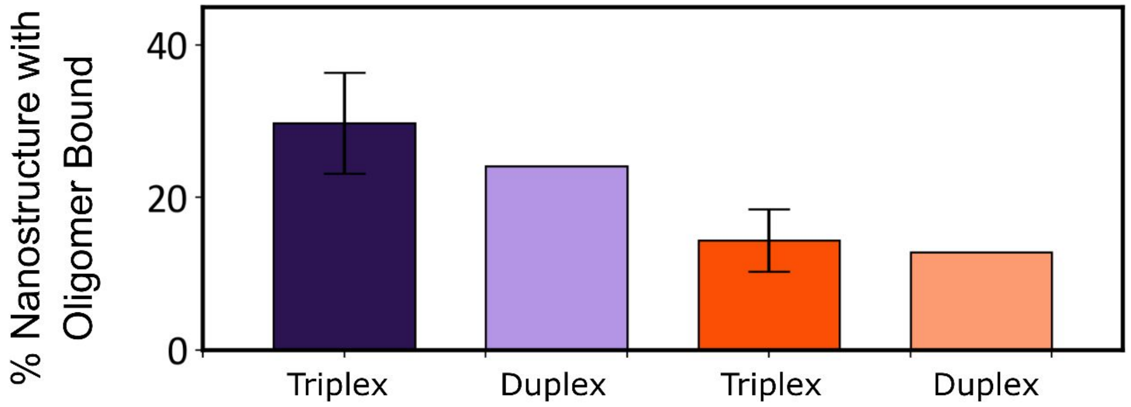

Figure S5. Comparison of Duplex and Triplex measurements. The samples that were measured in duplex show similar % of nanostructure with protein bound highlighting that monomer interchange is unlikely. The fraction of events with an oligomer bound to the DNA barcode; triplexed DMSO (purple) (N= 114, SD=6.62), duplexed DMSO light purple (N=54), triplexed I3.08 (orange) (N=90, SD=4.07), duplexed I3.08 (light orange) (N=39).

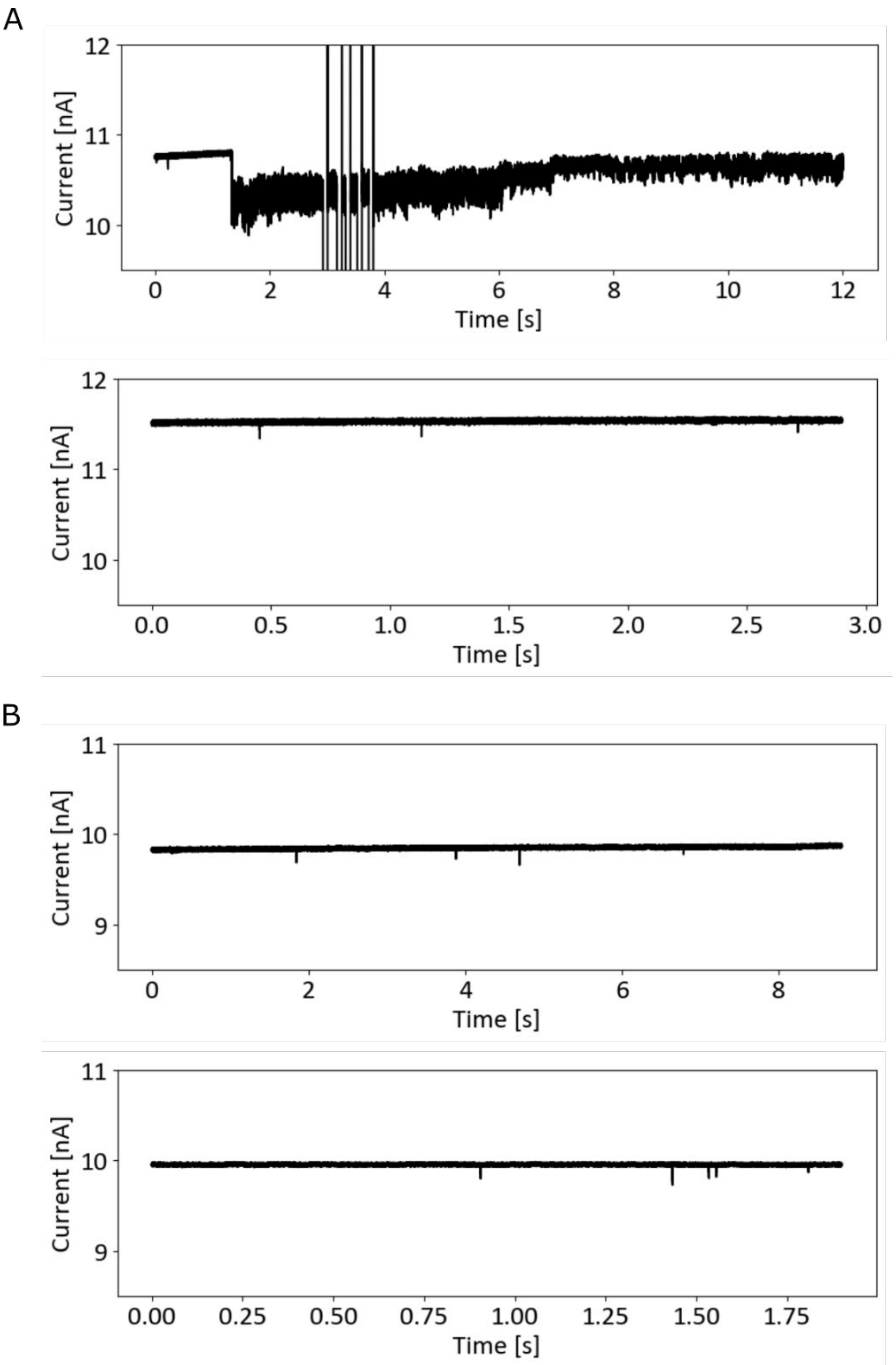

Figure S6. Nanopore traces with and without presence of Anle-138b. **(A)** Raw current of the nanopore trace with Anle-138b shows that upon mixture and measurement in the nanopore after 1 second, a lot of noise is created. After 3 min (trace below) the noise level resumes back to normal. The vertical lines represent kick outs to remove protein from clogging the pore. **(B)** Raw current

trace without Anle-138b shows similar noise and baseline both upon mixture and measurement in the pore and after 3 min of measurement.

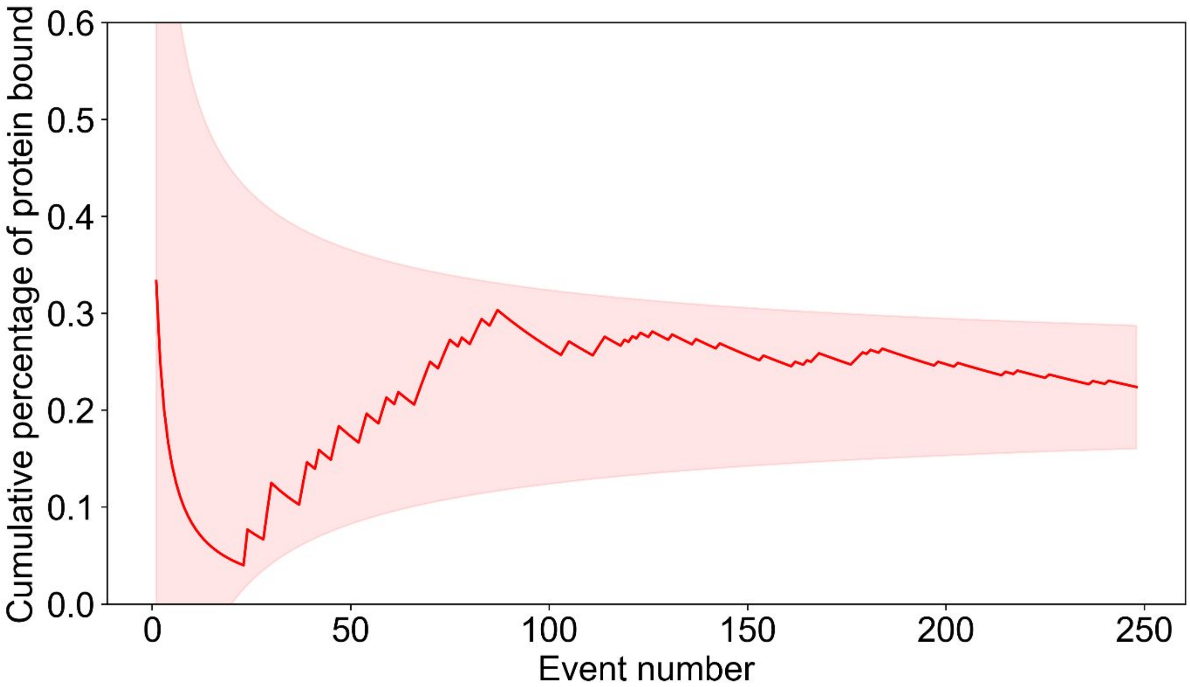

Figure S7. Cumulative percentage of events with clear barcode and protein bound as measured in 4 M LiCl. The number of events with the protein bound remains the same for the stabilized oligomer sample over the course of the 1 h measurement time. N=250 because the original trace was filtered to remove folded or knotted events with unreadable barcodes. The error fluctuation represents 1σ deviation. This becomes smaller as the number of events increases. The increase in number of events corresponds to measurement time.

The following replacements are made to create the “1” bits in the barcode portion of the nanostructure as previously shown and used in **Figure 2**[4].

First bit

Replace oligos 26,27,28,29,30,31 and 32

CTGAAAGCGTAAGAATACGTGGCACAGACAATATTTTTGAATGGCT
ACATCACTTGTCCTCTTTTGAGGAACAAGTTTTCTTGTCCTGAGTAGA
AGAACTCAAATCCTCTTTTGAGGAACAAGTTTTCTTGTCTATCGGCCT
TGCTGGTAATTCCTCTTTTGAGGAACAAGTTTTCTTGTATCCAGAACA
ATATTACCGCTCCTCTTTTGAGGAACAAGTTTTCTTGTCAGCCATTGC
AACAGGAAAATCCTCTTTTGAGGAACAAGTTTTCTTGTACGCTCATGG
AAATACCTACTCCTCTTTTGAGGAACAAGTTTTCTTGTATTTTGACGC
TCAATCGTCTTCCTCTTTTGAGGAACAAGTTTTCTTGTGAAATGGATT
ATTTACATTGTCCTCTTTTGAGGAACAAGTTTTCTTGTGCAGATTCAC
CAGTCACACGTCCTCTTTTGAGGAACAAGTTTTCTTGTACCAGTAATA
AAAGGGACATTCCTCTTTTGAGGAACAAGTTTTCTTGTTCTGGCCAAC
AGAGATAGAATCCTCTTTTGAGGAACAAGTTTTCTTGTCCCTTCTGAC

Second bit

Replace oligos 40,41,42,43,44,45 and 46

AATATAATCCTGATTGTTTGGATTATACTTCTGAATAATGGAAGGG
CACTAACAACTCCTCTTTTGAGGAACAAGTTTTCTTGTTAATAGATTA
GAGCCGTCAATCCTCTTTTGAGGAACAAGTTTTCTTGTTAGATAATAC
ATTTGAGGATTCCTCTTTTGAGGAACAAGTTTTCTTGTTTAGAAGTAT
TAGACTTTACTCCTCTTTTGAGGAACAAGTTTTCTTGTAAACAATTCG
ACAACTCGTATCCTCTTTTGAGGAACAAGTTTTCTTGTTTAAATCCTT
TGCCCGAACGTCCTCTTTTGAGGAACAAGTTTTCTTGTTTATTAATTT
TAAAAGTTTGTCCTCTTTTGAGGAACAAGTTTTCTTGTAGTAACATTA
TCATTTTGCGTCCTCTTTTGAGGAACAAGTTTTCTTGTGAACAAAGAA
ACCACCAGAATCCTCTTTTGAGGAACAAGTTTTCTTGTGGAGCGGAAT
TATCATCATATCCTCTTTTGAGGAACAAGTTTTCTTGTTTCCTGATTA
TCAGATGATGTCCTCTTTTGAGGAACAAGTTTTCTTGTGCAATTCATC

Third bit

Replace oligos 54,55,56,57,58,59 and 60

TGTAAATGCTGATGCAAATCCAATCGCAAGACAAAGAACGCGAGAA
TACCTTTTTTTCCTCTTTTGAGGAACAAGTTTTCTTGTAATGGAAACA
GTACATAAATTCCTCTTTTGAGGAACAAGTTTTCTTGTCAATATATGT
GAGTGAATAATCCTCTTTTGAGGAACAAGTTTTCTTGTCCTTGCTTCT
GTAAATCGTCTCCTCTTTTGAGGAACAAGTTTTCTTGTGCTATTAATT
AATTTTCCCTTCCTCTTTTGAGGAACAAGTTTTCTTGTTAGAATCCTT

GAAAACATAGTCCTCTTTTGAGGAACAAGTTTTCTTGTCGATAGCTTA
GATTAAGACGTCCTCTTTTGAGGAACAAGTTTTCTTGTCTGAGAAGAG
TCAATAGTGATCCTCTTTTGAGGAACAAGTTTTCTTGTATTTATCAAA
ATCATAGGTCTCCTCTTTTGAGGAACAAGTTTTCTTGTTGAGAGACTA
CCTTTTTAACTCCTCTTTTGAGGAACAAGTTTTCTTGTCTCCGGCTTA
GGTTGGGTTATCCTCTTTTGAGGAACAAGTTTTCTTGTTATAACTATA

Fourth bit

Replace oligos 68,69,70,71,72,73 and 74

TCATCGAGAACAAGCAAGCCGTTTTTATTTTCATCGTAGGAATCAT
AGAATATAAATCCTCTTTTGAGGAACAAGTTTTCTTGTGTACCGACAA
AAGGTAAAGTTCCTCTTTTGAGGAACAAGTTTTCTTGTAATTCTGTCC
AGACGACGACTCCTCTTTTGAGGAACAAGTTTTCTTGTAATAAACAAC
ATGTTCAGCTTCCTCTTTTGAGGAACAAGTTTTCTTGTAATGCAGAAC
GCGCCTGTTTTCCTCTTTTGAGGAACAAGTTTTCTTGTATCAACAATA
GATAAGTCCTTCCTCTTTTGAGGAACAAGTTTTCTTGTGAACAAGAAA
AATAATATCCTCCTCTTTTGAGGAACAAGTTTTCTTGTCATCCTAATT
TACGAGCATGTCCTCTTTTGAGGAACAAGTTTTCTTGTTAGAAACCAA
TCAATAATCGTCCTCTTTTGAGGAACAAGTTTTCTTGTGCTGTCTTTC
CTTATCATTCTCCTCTTTTGAGGAACAAGTTTTCTTGTCAAGAACGGG
TATTAAACCATCCTCTTTTGAGGAACAAGTTTTCTTGTAGTACCGCAC

Fifth bit

Replace oligos 82,83,84,85,86,87 and 88

AGATAGCCGAACAAAGTTACCAGAAGGAAACCGAGGAAACGCAATA
AAAAATGAAATCCTCTTTTGAGGAACAAGTTTTCTTGTATAGCAGCCT
TTACAGAGAGTCCTCTTTTGAGGAACAAGTTTTCTTGTAATAACATAA
AAACAGGGAATCCTCTTTTGAGGAACAAGTTTTCTTGTGCGCATTAGA
CGGGAGAATTTCCTCTTTTGAGGAACAAGTTTTCTTGTAACTGAACAC
CCTGAACAAATCCTCTTTTGAGGAACAAGTTTTCTTGTGTCAGAGGGT
AATTGAGCGCTCCTCTTTTGAGGAACAAGTTTTCTTGTTAATATCAGA
GAGATAACCCTCCTCTTTTGAGGAACAAGTTTTCTTGTACAAGAATTG
AGTTAAGCCCTCCTCTTTTGAGGAACAAGTTTTCTTGTAATAATAAGA
GCAAGAAACATCCTCTTTTGAGGAACAAGTTTTCTTGTATGAAATAGC
AATAGCTATCTCCTCTTTTGAGGAACAAGTTTTCTTGTTTACCGAAGC
CCTTTTTAAGTCCTCTTTTGAGGAACAAGTTTTCTTGTAAAAGTAAGC

Table S2. DNA Dumbell Bits

142	GATGGTTTAATTTCAACTTTAATCATTGTGAATTACCT
143	actgactgactgactgactgaTTTATGCGATTTTAAGAACTGGCTCATTATACCAGTCAGG
DBCO	tcagtcagtcagtcagtcagt*DBCO*

Table S3. DNA overhang sequences

Supplementary References

(1) Le Guilloux, V.; Schmidtke, P.; Tuffery, P. Fpocket: an open source platform for ligand pocket detection. *BMC Bioinformatics* **2009**, *10*, 168.

(2) Sormanni, P.; Aprile, F. A.; Vendruscolo, M. The CamSol method of rational design of protein mutants with enhanced solubility. *Journal of molecular biology* **2015**, *427* (2), 478-490.

(3) Horne, R. I.; Andrzejewska, E.; Alam, P.; Brotzakis, Z. F.; Srivastava, A.; Aubert, A.; Nowinska, M.; Gregory, R. C.; Staats, R.; Possenti, A. Discovery of Potent Inhibitors of α-Synuclein Aggregation Using Structure-Based Iterative Learning. *bioRxiv* **2021**, 2021.2011. 2010.468009.

(4) Bell, N. A.; Keyser, U. F. Digitally encoded DNA nanostructures for multiplexed, single-molecule protein sensing with nanopores. *Nature nanotechnology* **2016**, *11* (7), 645.
